# Suicides in France

**Published:** 1847-11

**Authors:** 


*Suicides in France.*—The report of the criminal justices of France, for the year 1845, contains the following details respecting the frequency of suicide, the means employed, and the motives. Of the number of deaths of the cause of which there has been suspicion, 11,049, there have been 3084 by suicide. The number of suicides in 1845 exceeds by 111 that of 1844, and by only sixty-four that of 1843. Of the 3084 suicides, 2332 were males, 725 females. Sixteen of the males and four of the females had not attained their sixteenth year; and among the number we find children of seven, eight, and ten years of age; 123 were between sixteen and twenty-one, 462 between twenty-one and thirty, 1201 between thirty and fifty, 945 between fifty and seventy, 203 between seventy and eighty, and forty-one above eighty. If we look to the relative number of suicides in the different months, we find 922 during the three summer months, June, July, and August; 861 during the three months of spring, March, April, and May; 756 in the three autumnal months, September, October, and November; lastly, only 545 in the three winter months, December, January, and February. The most common modes of suicide are hanging and drowning. 1110 suicides had recourse to the first of these ways, in 1845; 995 to drowning; 432 despatched themselves by firearms; whilst 213 asphyxiated themselves by the vapour of charcoal; this last named plan is most common in the Department of the Seine. The motives of suicide resemble those of other years: crosses in love, jealousy, the consequence of debauchery, misery, reverses of fortune, domestic troubles, the desire to rid themselves from physical sufferings, are the most common—*Ibid.*

